# Rapid Inactivation of Proteins by Rapamycin-Induced Rerouting to Mitochondria

**DOI:** 10.1016/j.devcel.2009.12.015

**Published:** 2010-02-16

**Authors:** Margaret S. Robinson, Daniela A. Sahlender, Samuel D. Foster

**Affiliations:** 1 University of Cambridge, Cambridge Institute for Medical Research, Cambridge CB2 0XY, UK

**Keywords:** CELLBIO, PROTEINS

## Abstract

We have developed a method for rapidly inactivating proteins with rapamycin-induced heterodimerization. Cells were stably transfected with siRNA-resistant, FKBP-tagged subunits of the adaptor protein (AP) complexes of clathrin-coated vesicles (CCVs), together with an FKBP and rapamycin-binding domain-containing construct with a mitochondrial targeting signal. Knocking down the endogenous subunit with siRNA, and then adding rapamycin, caused the APs to be rerouted to mitochondria within seconds. Rerouting AP-2 to mitochondria effectively abolished clathrin-mediated endocytosis of transferrin. In cells with rerouted AP-1, endocytosed cation-independent mannose 6-phosphate receptor (CIMPR) accumulated in a peripheral compartment, and isolated CCVs had reduced levels of CIMPR, but normal levels of the lysosomal hydrolase DNase II. Both observations support a role for AP-1 in retrograde trafficking. This type of approach, which we call a “knocksideways,” should be widely applicable as a means of inactivating proteins with a time scale of seconds or minutes rather than days.

## Graphical abstract

**Graphical Abstract:**

**Figure:**
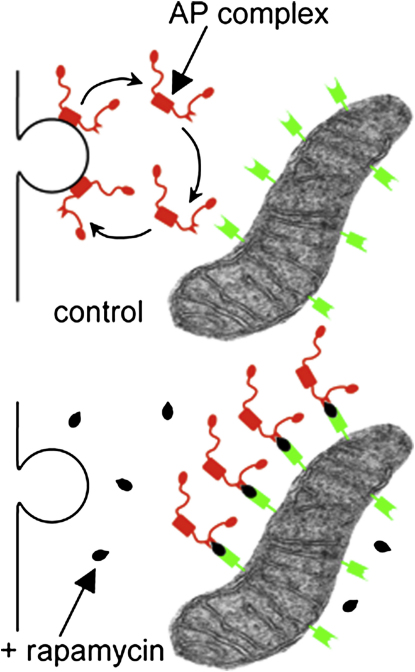

**Highlights:**

► We have developed a way of getting rid of proteins quickly (i.e., minutes not days) ► This approach provides new insights into the function of the coat protein AP-1

## Introduction

Establishing the function of a protein can be difficult. Even proteins that have been extensively characterized may remain elusive when it comes to finding out precisely what they do. A case in point is the adaptor protein (AP)-1 complex, which packages cargo proteins into clathrin-coated vesicles (CCVs) budding from intracellular membranes. In spite of numerous studies on the phenotype of cells and organisms with AP-1 either deleted or depleted, there is still some uncertainty about the function of AP-1, in particular whether it facilitates trafficking from the *trans*-Golgi network (TGN) to endosomes, from endosomes to the TGN, or both (Robinson, 2004). Theoretically, it should be possible to determine the directionality of AP-1 trafficking by looking for changes in the steady-state distribution of cargo proteins that depend upon AP-1 for packaging. However, AP-1 knockout and knockdown studies have produced conflicting results. In some experiments, cargo proteins were found to accumulate in endosomal (Meyer et al., 2000) or postendosomal (Foote and Nothwehr, 2006) compartments, while, in others, they accumulated in the Golgi region (Canuel et al., 2008) or at the plasma membrane (Lubben et al., 2007).

The major problem with gene knockouts and knockdowns is that one cannot assay the phenotype immediately, because the cell still contains “old” protein that needs to be degraded and/or diluted. In the case of long-lived proteins like AP-1 subunits, it can take several days for the protein concentration to drop to <10% of control levels. During this time, the sorting of cargo proteins is likely to become less and less efficient as the levels of AP-1 diminish. Some of these cargo proteins may be components of the trafficking machinery, such as SNAREs (Peden et al., 2001). Thus, by the time the cells are analyzed, other membrane proteins may be missorted, not as a direct consequence of the AP-1 knockdown, but as a secondary effect, due to the mislocalization of machinery. Another potential problem is that knocking down one trafficking pathway may lead to upregulation of other compensatory pathways (Damke et al., 1995). Therefore, more rapid methods are needed in order to investigate the immediate consequences of inactivating AP-1. Ideally, such methods should be adaptable enough that they could be applied to other proteins as well.

The usual strategy for inactivating a protein quickly is to add a small molecule inhibitor that can readily cross the plasma membrane. So far, only a tiny percentage of proteins can be targeted by specific drugs, but, over the past 15–20 years, a number of cell biologists have carried out small-molecule screens to try to find new inhibitors of membrane traffic pathways (Takizawa et al., 1993; Macia et al., 2006). One such screen identified a family of compounds that interfere with AP-1-dependent trafficking between the TGN and endosomes in yeast (Duncan et al., 2007). However, the precise mechanism of action of these compounds is not clear, and, when used on mammalian cells, the only clear phenotype was an increase in the perinuclear localization of AP-1 by immunofluorescence.

An alternative strategy is to modify the protein of interest so that it can be targeted by an existing drug. For instance, rapamycin is a drug that can be used to form heterodimers between any two proteins, as long as they are in the same compartment and contain rapamycin-binding domains. Normally, rapamycin binds first to FKBP12, a prolyl isomerase, and then the rapamycin-FKBP12 complex binds to mammalian target of rapamycin (mTOR), a kinase. When the FKBP domain from FKBP12 and the FKBP and rapamycin-binding (FRB) domain from mTOR are transplanted onto other proteins, these proteins will also dimerize in the presence of rapamycin. Rapamycin-induced heterodimerization has been used in a number of cell biological studies to address questions such as whether ER and Golgi membranes fuse during mitosis (Pecot and Malhotra, 2004), and how phosphoinositides contribute to clathrin-mediated endocytosis (Zoncu et al., 2007) and intracellular trafficking (Zoncu et al., 2009). Two recent studies in yeast used rapamycin to inactivate nuclear proteins by sequestering them in the cytoplasm (Haruki et al., 2008; Geda et al., 2008). Rapamycin and related compounds have also been used to attach signaling molecules to the plasma membrane, and thus constitutively activate them (Spencer et al., 1995; Belshaw et al., 1996; Graef et al., 1997; Inoue et al., 2005). However, so far, there is no rapamycin-based method that can be used to inactivate a wide range of proteins with different subcellular distributions.

Another drug that binds to the FKBP domain is FK506, and an artificial dimer of FK506, called FK1012, can be used to form homodimers between FKBP-tagged proteins. Attaching an FKBP domain to clathrin and then adding FK1012 to transfected cells has been shown to cross-link the clathrin and disrupt the normal cycle of clathrin assembly/disassembly, inhibiting clathrin-mediated endocytosis by ∼70% (Moskowitz et al., 2003). However, because this method can only be used to inactivate proteins that naturally self-assemble into oligomers, it is not very adaptable. Probably the most wide-ranging strategy for perturbing proteins pharmacologically is one that makes use of a modified FKBP or FRB domain, which renders tagged proteins unstable. The proteins can be protected by the addition of a synthetic ligand, and the ligand can then be withdrawn, causing the proteins to be degraded. The advantage of this method is that it can be applied to many different types of proteins, and it has now been successfully used on whole animals as well as on cultured cells (Stankunas et al., 2003; Banaszynski et al., 2006, 2008). The disadvantage of this method is that it is relatively slow: protein degradation after ligand withdrawal has a t_1/2_ of at least 1 hr, during which time extensive trafficking (and other activities) can occur.

We set out to develop a method that would be both rapid and versatile. The idea was to relocate proteins such as AP-1 to a subcellular compartment that does not participate in either the secretory or the endocytic pathway. To this end, we made use of a technique developed by workers in the cytoskeleton field, in which proteins are recruited onto the mitochondrial outer membrane (Bear et al., 2000). This is normally done by attaching a mitochondrial targeting signal to a protein that has a number of different binding partners. The binding partners accumulate on mitochondria, where they are unavailable for their usual role. The cells seem to tolerate the presence of “foreign” proteins on their mitochondria surprisingly well, and this method has been used to provide information not only about protein-protein interactions (Kessels and Qualmann, 2002), but also about protein function (Dwivedy et al., 2007). However, because the mitochondrial construct first has to be synthesized by the cells, the phenotype cannot be analyzed until hours or days after transfection.

In the present study, we have combined mitochondrial rerouting with rapamycin-induced heterodimerization in order to acutely sequester proteins away from their normal site of action. Two proteins were chosen for these experiments: the γ subunit of the AP-1 adaptor complex and, for comparison, the α subunit of the AP-2 adaptor complex. Like AP-1, AP-2 is an adaptor for CCVs, but it participates in endocytosis rather than intracellular trafficking. Its role is much better understood than that of AP-1, and there are more reagents and assays available. Here we show that rapamycin-induced rerouting to mitochondria is extremely rapid, and that it can provide new insights into both protein dynamics and protein function.

## Results

### FKBP-Tagged AP Complexes Behave Normally

**Figure 1:**
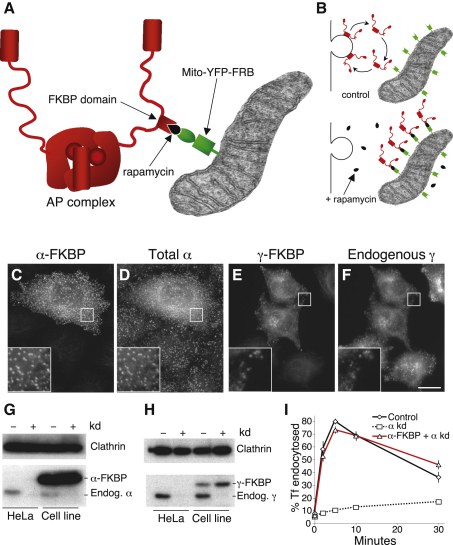
FKBP-Tagged AP Complexes (A and B) Schematic diagrams of the strategy that was used. (C–F) Stable cell lines expressing either α-FKBP (C and D) or γ-FKBP (E and F) were mixed with nontransfected cells and double labeled for the construct and either total α or endogenous γ (using a species-specific antibody). The insets show higher magnification views of the areas inside the boxes. Scale bar, 20 μm. (G and H) Homogenates of control HeLa cells and cell lines expressing either α-FKBP (G) or γ-FKBP (H) were depleted of the endogenous subunit, and western blots were probed with antibodies that recognize both the endogenous protein and the tagged construct. (I) Control and α-FKBP-expressing cells were depleted of endogenous α using siRNA, and endocytosis of prebound ^125^I-labeled transferrin (Tf) was measured. Data are presented as the means (±SE) from three independent experiments.

The FKBP domain was inserted into the linker region of siRNA-resistant, epitope-tagged versions of the α subunit of AP-2 and the γ subunit of AP-1 (Figures 1A and 1B), and the constructs were stably transfected into HeLa cells. Immunofluorescence showed that both constructs colocalized with the endogenous and/or total protein (Figures 1C–1F), and siRNA knockdowns showed that, in both cases, only the endogenous protein was depleted by the siRNA (Figures 1G and 1H). To ensure that the insertion of an FKBP domain does not inactivate the AP subunit, we used a well-established assay for AP-2 function (Figure 1I). Endocytosis of radioiodinated transferrin was measured in three populations of cells: untreated cells, cells depleted of endogenous α, and the α-FKBP-expressing cell line depleted of endogenous α. The α-FKBP construct efficiently rescued the knockdown phenotype, indicating that it is fully functional.

### Rapamycin Reroutes Tagged APs to Mitochondria

**Figure 2:**
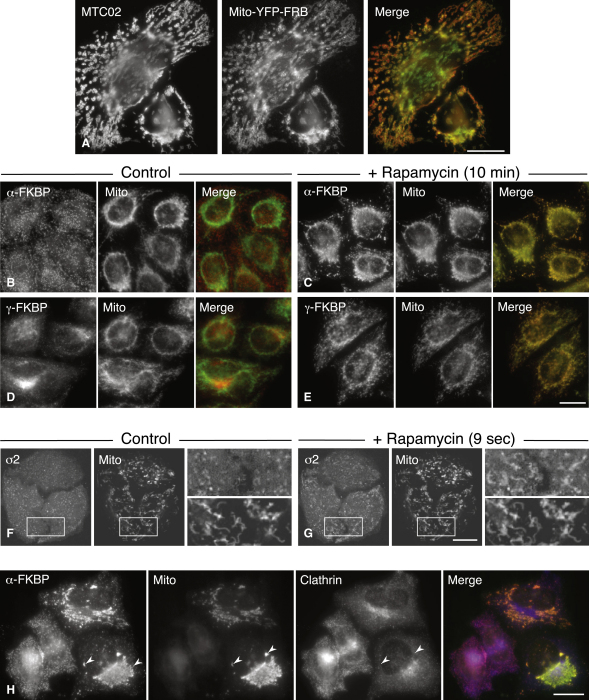
Localization of Constructs and Effect of Rapamycin (A) Cells expressing Mito-YFP-FRB were labeled with an antibody against a mitochondrial marker, MTC02. (B–E) Stable cell lines coexpressing either α-FKBP (B and C) or γ-FKBP (D and E) together with Mito-YFP-FRB were treated with 200 nM rapamycin for either 0 (B and D) or 10 (C and E) min. Electron micrographs of the same conditions are shown in Figure S1. (F and G) Live-cell imaging of cells coexpressing α-FKBP and Mito-YFP-FRB, and transiently transfected with mCherry-tagged σ2. See Movie S1 for the video link. Rapamycin was added to the cells at time 0 (F), which corresponds to 1 min in the video; the frames in (G) show the same cells 9 s later. The panels on the right of (F) and (G) are higher magnification views of the areas inside the boxes. (H) Localization of clathrin in cells with rerouted AP-2. Mixed populations of cells, expressing either α-FKBP only, or coexpressing α-FKBP and Mito-YFP-FRB, were depleted of endogenous α, treated with 200 nM rapamycin for 10 min, and triple labeled for clathrin. Other triple-labeled images are shown in Figure S2. Scale bars, 20 μm.

Next, we designed a construct called Mito-YFP-FRB (Figures 1A and 1B), consisting of the import signal of the yeast mitochondrial outer membrane protein Tom70p, which has been shown to work in mammalian cells (Kessels and Qualmann, 2002), followed by YFP as a reporter and the FRB domain. Figure 2A demonstrates that Mito-YFP-FRB is correctly targeted to mitochondria, because it colocalizes with the mitochondrial marker MTC02. We then generated clonal cell lines that coexpress either α-FKBP or γ-FKBP together with Mito-YFP-FRB. In the absence of rapamycin, there was no colocalization between the two constructs (Figures 2B and 2D). However, addition of rapamycin for 10 min caused the adaptors to be almost completely rerouted to mitochondria (Figures 2C and 2E; see Figure S1 available online).

To investigate the time course of the rapamycin response, we transiently transfected our α-FKBP/Mito-YFP-FRB cell line with mCherry-tagged σ2 (another subunit of the AP-2 complex), and then carried out live cell imaging. Figure 2F shows cells at the time of addition of rapamycin, and Figure 2G shows the cells 9 s later. It is clear that the rapamycin response is already well underway; indeed, we could see rerouting just 3 s after addition of the drug, which was as quickly as we could capture the images (Movie S1).

What happens to the rest of the CCV machinery in cells with rerouted AP complexes? To compare the labeling patterns of other CCV-associated proteins, we knocked down the endogenous AP subunits in both singly and doubly transfected cell lines, and then mixed the cells together and added rapamycin. Triple labeling showed that some CCV-associated proteins, such as Dab2 and epsinR, followed their respective APs onto mitochondria. However, other proteins, including clathrin, did not (Figure 2H; Figure S2), indicating that the cells do not attempt to form CCVs from mitochondrial membranes.

### Transferrin Uptake Is Blocked in Cells with Rerouted AP-2

**Figure 3:**
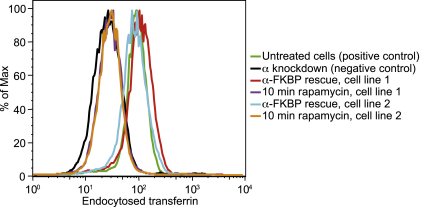
Assay for AP-2 Function Two different cell lines that stably express both α-FKBP and Mito-YFP-FRB were depleted of endogenous α and allowed to endocytose fluorescently labeled transferrin for 10 min, with or without first treating with rapamycin for 10 min. As a comparison, control cells, either left untreated or depleted of endogenous α, were also allowed to endocytose fluorescent transferrin for 10 min. After stripping off surface-bound transferrin, internalized transferrin was quantified by flow cytometry.

To examine the effects of rerouting on AP function, we looked first at AP-2. Transferrin uptake was quantified by flow cytometry in two different cell lines that coexpress α-FKBP and Mito-YFP-FRB, which had been depleted of endogenous α with siRNA. Addition of rapamycin for 10 min produced a similar inhibition of transferrin uptake to depleting AP-2 in control cells (Figure 3). Thus, at least in the case of AP-2, rapamycin-induced rerouting works just as well as a conventional knockdown. However, the procedure takes 10 min instead of 4 days, which means that we can investigate the early consequences of protein inactivation.

### CIMPR Is Mislocalized in Cells with Rerouted AP-1

Having established that the rapamycin system works well on AP-2, we went on to investigate AP-1, looking first at the localization of the cation-independent mannose 6-phosphate receptor (CIMPR). The CIMPR is a receptor for lysosomal hydrolases, which binds the hydrolases in the TGN, delivers them to endosomes, and then returns to the TGN for another round. At steady state, the CIMPR normally localizes mainly to the TGN and/or endosomes (the relative levels in each compartment depend on cell type), but it occasionally travels out to the plasma membrane, where it is rapidly retrieved by clathrin-mediated endocytosis. In cells from AP-1 knockout mice, the CIMPR was found to accumulate in a peripheral endosomal compartment, supporting a role for AP-1 in retrograde traffic (Meyer et al., 2000). However, because the cells had many days to adjust to the lack of AP-1, one cannot rule out the possibility that this phenotype was an indirect effect of the knockout.

**Figure 4:**
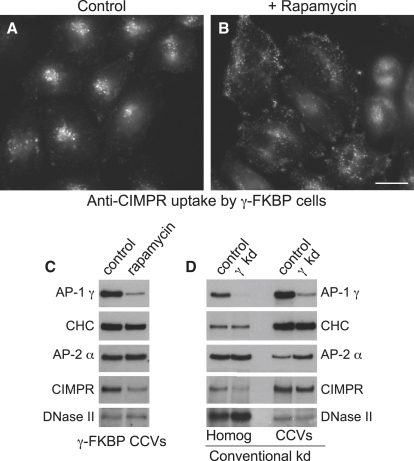
Assays for AP-1 Function (A and B) Cells coexpressing γ-FKBP and Mito-YFP-FRB were depleted of endogenous γ with siRNA, and incubated with anti-CIMPR for 45 min at 37°C, either without (A) or with (B) rapamycin. See also Figure S3. Scale bar, 20 μm. (C) Cells coexpressing γ-FKBP and Mito-YFP-FRB were depleted of endogenous γ with siRNA. Half of the cells were treated with rapamycin for 10 min before homogenization, then CCVs were isolated and western blots probed with the indicated antibodies. (D) Blots of whole-cell homogenates and CCV preparations from a conventional AP-1 γ knockdown, probed with the same antibodies as in (C).

Cells coexpressing γ-FKBP and Mito-YFP-FRB were treated with rapamycin for various lengths of time. For some experiments, the cells were fed an antibody against the CIMPR during the treatment. The cells were then fixed, and the localization of either endocytosed anti-CIMPR or total CIMPR was investigated by immunofluorescence. Rapamycin caused both the internalized antibody and the total receptor to accumulate in peripheral rather than juxtanuclear membranes, but this was particularly striking for the internalized antibody (Figures 4A and 4B; Figure S3). We saw a similar but less dramatic phenotype when we carried out a conventional AP-1 knockdown, while, in control cells, rapamycin treatment for the same length of time had no effect on CIMPR or antibody localization (Figure S3).

### CIMPR Is Depleted in CCVs from Cells with Rerouted AP-1, but DNase II Is Not

Although the experiments described above support a role for AP-1 in retrograde traffic, the effects of the drug are not immediately apparent (the cells in Figure 4 and Figure S3 were incubated with rapamycin for 45 min). This is because it takes some time to change the steady-state distribution of a membrane protein by interfering with its packaging into CCVs. Only a small fraction of the protein is present in CCVs at any one time, so one has to wait for multiple rounds of CCV budding and fusion before a change in the overall localization of the protein is perceptible. Therefore, we used another method to look for mislocalization: we isolated CCVs from the cells, either with or without first treating them with rapamycin. This gives us a snapshot of the CCVs at the moment when the cells are homogenized, so it is ideally suited to the present study, because we can analyze the phenotype of the cells within minutes of adding the drug.

Cells coexpressing γ-FKBP and Mito-YFP-FRB were first treated with siRNA to knock down endogenous γ. Half of the dishes were then incubated with rapamycin for 10 min before homogenization. CCVs were isolated and western blotting was used to look for differences in the relative abundance of various CCV components. A representative preparation is shown in Figure 4C.

In the rapamycin-treated cells, the AP-1 complex was lost from CCVs, and there was also a subtle but reproducible loss of clathrin, while AP-2 levels remained constant. The CIMPR was also depleted in CCVs prepared from cells with rerouted AP-1. In contrast, DNase II, a hydrolase that we have previously shown to be a bona fide CCV cargo protein (Borner et al., 2006), and which is known to contain mannose 6-phosphate (Sleat et al., 2005), was unaffected by rapamycin, indicating that its packaging is not dependent on AP-1. Although it will be important to follow up this observation by quantifying the levels of other hydrolases in the CCV preparation by comparative proteomics, the DNase II result is likely to be representative. The loss of the CIMPR from the preparation, with no concomitant loss of DNase II, supports a role for AP-1 in the retrograde trafficking of the empty receptor, rather than in the anterograde trafficking of the receptor-hydrolase complex.

How does rapamycin-induced rerouting of AP-1 compare with a conventional knockdown? Figure 4D shows CCVs isolated from cells treated with siRNA for 4 days to deplete AP-1 γ, together with a matched control from the same experiment. Although there is less CIMPR in the CCVs after the knockdown, there is also less CIMPR in the homogenate, presumably because it is degraded more rapidly, and this makes the difference in the CCVs less clear cut than in the rapamycin experiment. DNase II is also somewhat reduced in the CCVs, but we suspect that this is an indirect consequence of mislocalization of the CIMPR. In contrast, AP-2 is actually increased in the CCVs, possibly reflecting an attempt by the cells to compensate for the loss of AP-1. Together, these results show that data obtained from a conventional knockdown can sometimes be difficult to interpret, and that acute depletion can provide insights into protein function that are not apparent when proteins are depleted gradually.

## Discussion

We have developed a strategy for investigating protein function, in which we use rapamycin to trap the proteins on mitochondria. Like all scientific methods, our strategy builds on techniques developed by other researchers: rapamycin-mediated heterodimerization and mitochondrial mislocalization. However, to the best of our knowledge, this is the first time that the two techniques have been combined, and the synthesis of these two approaches allows us to take advantage of the rapidity and inducibility of the rapamycin system, together with the availability of mitochondria as a platform for sequestering proteins away from their normal site of action. Because rapamycin-induced rerouting is similar in some respects to a knockout or a knockdown, but is much more rapid and puts the proteins in a strange location instead of destroying them, we propose a name for it that (in British English) means “take by surprise”: a knocksideways.

In the case of AP-2, the knocksideways phenotype is very similar to that of a conventional knockdown. However, in the case of AP-1, mislocalization of endocytosed anti-CIMPR and changes in the protein composition of CCVs are more pronounced in knocksideways cells than in a conventional knockdown. This is most likely because AP-1 is depleted rapidly in the knocksideways cells, but only very gradually in the knockdown, giving the cells a chance to adjust. One potential caveat is that the AP-1 binding partner, epsinR, and possibly other proteins as well, follow AP-1 onto mitochondria in the rapamycin-treated knocksideways cells (Figure S2), so we cannot formally rule out the possibility that the loss of one or more other proteins also contributes to the knocksideways phenotype. Nevertheless, both the antibody uptake assay and the CCV isolation assay support a role for AP-1 in the TGN-to-endosome pathway, adding to the weight of evidence implicating AP-1 in retrograde rather than anterograde trafficking (Bonifacino and Rojas, 2006).

How does the knocksideways approach compare with other methods for perturbing protein function? The time scale is approximately three to four orders of magnitude faster than an siRNA knockdown, and approximately one to two orders of magnitude faster than the only other drug-based method that can be applied to a wide range of proteins: chemically controlled degradation with a destabilizing FKBP domain (Banaszynski et al., 2006). The kinetics of rerouting reflect the speed with which adaptors cycle on and off membranes: fluorescence recovery after photobleaching experiments show that, at 37°C, bleached-coat proteins are replaced with a half-time of ∼10–20 s (Wu et al., 2003; Puertollano et al., 2003), and this occurs even in the absence of vesicle budding. Thus, rapamycin-induced rerouting occurs over a similar time frame as the transient protein-protein and protein-lipid interactions that take place inside the cell, which makes it ideal for studying this type of event in vivo.

Why are the adaptors recruited so readily onto mitochondria, instead of rebinding to the membrane they came from? Part of the reason must be that the interaction with mitochondria has a much higher affinity. The interaction between the FKBP domain and rapamycin, and between the FKBP-rapamycin complex and the FRB domain, both have a K_d_ of <1 nM (Bayle et al., 2006). In contrast, normal recruitment of adaptors onto membranes occurs through several low-to-moderate affinity interactions, typically in the low micromolar range (Owen et al., 2004). In addition, the FKBP-rapamycin-FRB interaction has a very slow off rate: dissociation of rapamycin from the FKBP domain has been reported to have a t_1/2_ of ∼17.5 hr (Hosoi et al., 1999). Mitochondria are present throughout the cell, so they would always be in close proximity to coat proteins when they dissociate from membranes, and, once an adaptor had bound to a mitochondrion, the interaction would be essentially irreversible and the adaptor would no longer be part of the cycling pool.

Although setting up the knocksideways system for a particular protein is fairly labor intensive, requiring doubly transfected cell lines and a robust knockdown-rescue system, the approach is one that could lend itself to many different types of proteins, as long as they are either cytosolic or transiently associated with membranes. Proteins in this category include other types of coats, such as COPI and COPII, which are difficult to study by siRNA knockdown, because they are essential for cell viability, as well as other proteins involved in membrane traffic, such as ARF and Rab GTPases, cytoskeletal motors, and tethering complexes. In addition, many proteins involved in cell signaling cycle back and forth between cytosol and membranes, and thus they too could be explored by the knocksideways method.

Although, so far, we have looked only at HeLa cells, mitochondrial rerouting could be used on other cell types, such as yeast and *Drosophila* cells, as well as on more complex organisms. We are currently in the process of making transgenic mice, which will constitutively express a mitochondrial targeting signal attached to a modified FRB domain, called FRB^∗^. FRB^∗^ has a point mutation that enables it to bind to a rapamycin analog, AP21967, which cannot bind the wild-type FRB domain (Bayle et al., 2006), thus circumventing any problems that might arise from inhibition of mTOR-mediated signaling. The plan is to mate these mice with knockin mice that have the FKBP domain inserted into their AP-1 γ gene, to generate a knocksideways mouse model. Although still in its early stages, this approach, if successful, should enable researchers to rapidly inactivate proteins of interest not only in cultured cells, but also in whole animals, and thus explore the role of the proteins in cell type-specific pathways.

## Experimental Procedures

### Constructs, Cell Lines, and Knock Downs

The α-FKBP construct was based on a pIRESneo2 plasmid encoding an siRNA-resistant form of α, which includes a brain-specific insert that acts as an epitope tag (Motley et al., 2006). The FKBP coding sequence was amplified by PCR and inserted into the linker region of α. The open reading frame was sequenced, the plasmid was transfected into HeLaM cells, and clonal cell lines were isolated as previously described (Motley et al., 2006).

For the γ-FKBP construct, a QuikChange mutagenesis kit (Stratagene) was used to introduce silent mutations into mouse γ cDNA to make it siRNA-resistant (see below) and to add a new restriction site, which was then used to insert the brain-specific epitope and the FKBP domain. After sequencing the open reading frame, the construct was moved into a retroviral vector, pLXIN, with a modified polylinker (a kind gift from A. Peden, CIMR). Virions were isolated from packaging cells and used to infect HeLaM cells, and stable cell lines were selected with G418.

The Mito-YFP-FRB construct was based on a pEYFP-FRB plasmid (kindly provided by O. Glebov, MRC Laboratory of Molecular Biology, Cambridge, UK). The N-terminal sorting signal of Tom70p was amplified by PCR from yeast genomic DNA and cloned into the plasmid, then the Mito-YFP-FRB coding sequence was moved into the retroviral vector pQCXIH (Clontech), which carries a hygromycin resistance gene, and stable cell lines were selected as above.

The siRNA targeting AP-2 α (α-2) has already been described (Motley et al., 2006). The γ-FKBP construct was made resistant to siRNA D-019183-02-0050 (Dharmacon) (γ-SP), which has the sequence, GAAGAUAGAAUUCACCUUU. Both siRNAs were used at a final concentration of 100 nM, with hits on Days 1 and 3, and assays carried out on Day 5, as previously described (Motley et al., 2003).

Rapamycin (Sigma) was prepared as a 1 mg/ml stock in ethanol and used at a concentration of 200 nM.

### Immunofluorescence and Western Blotting

Cells were prepared for immunofluorescence either by fixing with 3.7% paraformaldehyde and permeabilizing with 0.1% Triton X-100, or by fixing/permeabilizing with methanol at −20°C, as previously described (Motley et al., 2003). Images were acquired with a Zeiss Axiovert 200 inverted microscope and Improvision OpenLab software. Western blots were prepared as previously described (Borner et al., 2006), with cell homogenates, high-speed supernatants from CCV preparations, or isolated CCVs as samples (see below). Primary antibodies that had been made in house include rabbit antisera against clathrin heavy chain, AP-2 αC, AP-1 γ, epsinR, CIMPR, and the epitope used for tagging the AP subunits, A706–727 (Ball et al., 1995). Other primary antibodies include commercially available monoclonal antibodies against AP-1 γ (mAb100/3; Sigma), AP-2 α (BD), CIMPR (2G11; AbCam), and the mitochondrial protein MTC02 (AbCam), polyclonal anti-GFP and anti-DNase II (both from AbCam), and polyclonal anti-CALM and anti-Dab2 (both from Santa Cruz Biotechnology). The monoclonal antibodies against AP-2 α (AP.6) and clathrin heavy chain (X22) used for immunofluorescence were kind gifts from F. Brodsky (UCSF). The antibody against GGA2 was a kind gift from D. Brooks (Women's and Children's Hospital, North Adelaide, SA, Australia). Secondary antibodies for immunofluorescence were purchased from Invitrogen. Labeling on western blots was visualized with either ^125^I-protein A (Amersham) or HRP-conjugated secondary antibodies (Sigma) followed by chemiluminescence.

### Uptake Assays

Uptake of ^125^I-labeled transferrin was carried out as previously described (Motley et al., 2003), prebinding the transferrin to the cells at 4°C and then warming them up for various lengths of time. Surface-bound, internalized, and recycled counts were all quantified. The flow cytometry-based assay has also been described (Motley et al., 2006); briefly, cells were trypsinized and incubated with AlexaFluor 633-labeled transferrin at 4°C to allow binding to occur, then warmed to 37°C for 10 min and chilled again. Unbound and surface-bound label were both removed, and the cells were analyzed by flow cytometry with a BD FACSCalibur. Uptake of anti-CIMPR was carried out by diluting the antibody 1:5000 in tissue culture medium, inverting coverslips over drops of the antibody, and incubating at 37°C. Because there is relatively little CIMPR on the cell surface at steady state, incubations were normally carried out for 45 min. The cells were then fixed and labeled with secondary antibodies, as described above. To make sure that there was no bias in scoring the phenotypes, the slides were coded and scrambled before they were viewed.

### Live Cell Imaging

Stably transfected cells expressing both α-FKBP and Mito-YFP-FRB were transiently transfected with a plasmid encoding m-Cherry-tagged AP-2 σ2, constructed by moving the insert from pσ2EGFP-C1 (a kind gift from T. Kirchhausen, Harvard Medical School) into pmCherry-C1 (Clontech). Imaging was performed with a spinning disk confocal laser source mounted on a Zeiss AxioObserver Z1 inverted microscope and driven by Improvision Volocity Acquisition software. The images were collected every 3 s, and rapamycin was added as a 10× stock (i.e., 2 μM) after 1 min.

### Isolation of CCVs

Cells were grown until they formed confluent monolayers in 15 cm dishes. For the rapamycin experiments, two of the dishes were treated with 200 nM rapamycin for 10 min before homogenization, and two were left untreated. Ten independent preparations were carried out, using two different cell lines that coexpress γ-FKBP and Mito-YFP-FRB, with consistent results. The CCV protocol is based on differential centrifugation, and has been previously described (Hirst et al., 2004; Borner et al., 2006).

### Immuno-Electron Microscopy

For immunogold labeling, cells were fixed initially by adding an equal volume of freshly prepared 4% paraformaldehyde/0.4% gluteraldehyde in 0.25 M HEPES buffer (pH 7.4). After 2 min, the solution was removed and replaced by 2% paraformaldehyde/0.2% glutaraldehyde in 0.25 M HEPES (pH 7.4). Cells were incubated in this solution for 2 hr at room temperature and then further processed as previously described (Hirst et al., 2009). γ-FKBP and α-FKBP were detected with A706–727 (Ball et al., 1995), and Mito-YFP-FRB with anti-GFP. Double labeling was performed with protein A conjugated to colloidal gold (Utrecht University) as previously described (Hirst et al., 2009). Controls were carried out omitting the second antibody to ensure that blockage was complete, and varying the order in which the antibodies were added to ensure that the results were consistent. The grids were viewed with a Phillips CM 100 transmission electron microscope (Philips Electron Optics, Cambridge, UK) at an operating voltage of 80 kV.

## Supplemental Information

Document S1. Three Figures

Movie S1. Live cell imaging of rapamycin-induced rerouting of AP-2 to mitochondria See Figure 2 for still images.
