# Supplementary material for: Rapid Inactivation of Proteins by Rapamycin-Induced Rerouting to Mitochondria
Source: Dev Cell. 2010 Feb 16;18(2-3):324–31. doi: 10.1016/j.devcel.2009.12.015 (PMC2845799; doi:10.1016/j.devcel.2009.12.015)
Supplement: Document S1. Three Figures [file mmc1.pdf]

Developmental Cell *18*

Supplemental Information

Rapid Inactivation of Proteins

by Rapamycin-Induced Rerouting to Mitochondria

Margaret S. Robinson, Daniela A. Sahlender, and Samuel D. Foster

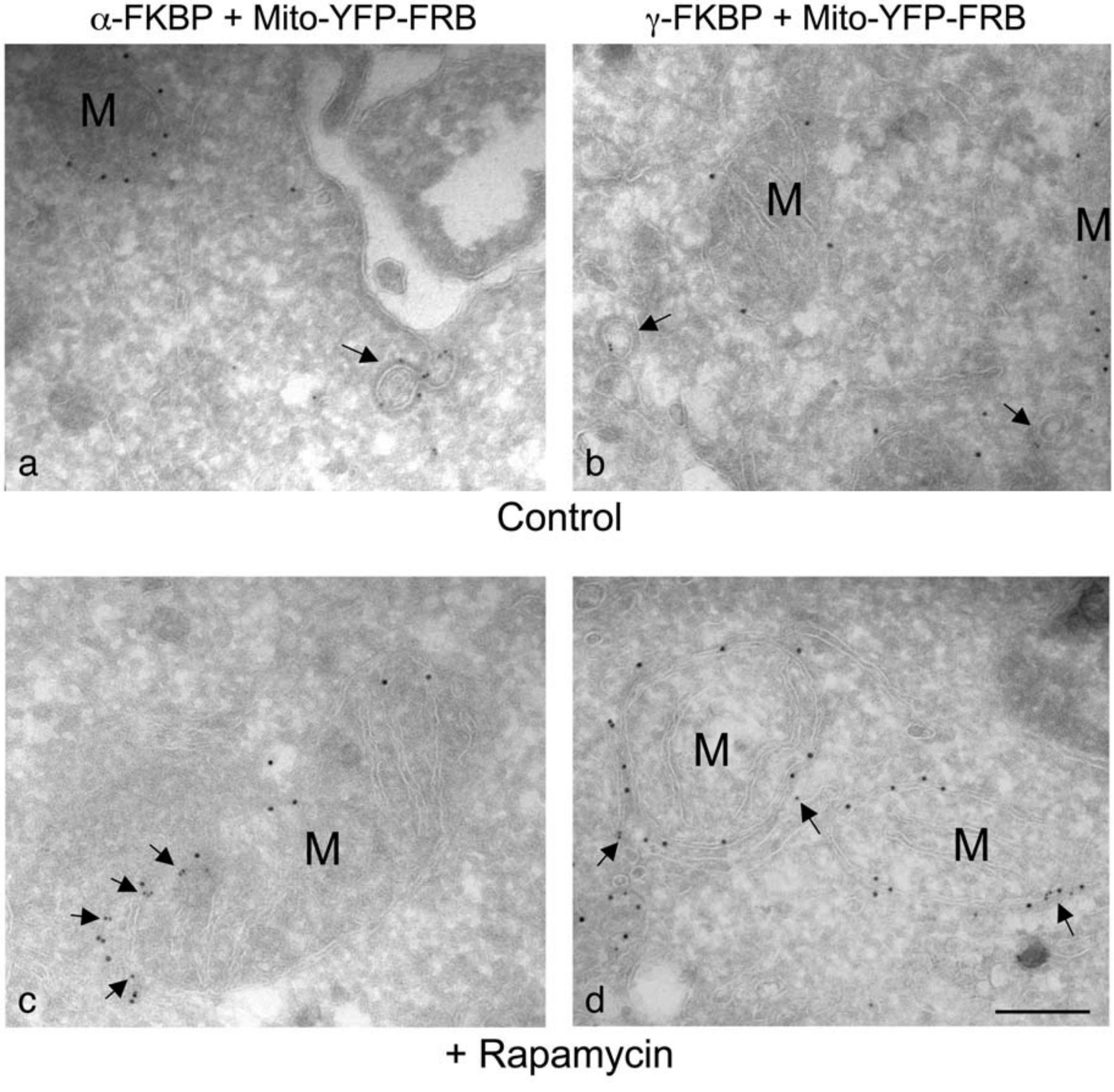

Figure S1. Immunogold labeling of APs rerouted onto mitochondria
Cells coexpressing either α-FKBP (a and c) or γ-FKBP (b and d) and Mito-YFP-FRB were depleted of the endogenous AP subunit and treated with 200 nM rapamycin for either 0 (a and b) or 10 (c and d) minutes. The cells were then fixed for electron microscopy, and frozen thin sections were double labeled with an antibody against the FKBP-tagged construct followed by 10 nm protein A-gold, and anti-GFP (which cross-reacts with Mito-YFP-FRB) followed by 15 nm protein A-gold. In the control cells (a and b), the 10 nm gold particles (indicated with arrows) decorate clathrin-coated budding profiles, associated with either the plasma membrane (a) or intracellular membranes (b). In the rapamycin-treated cells, the 10 nm gold particles are found on mitochondria (M). Scale bar: 200 nm.

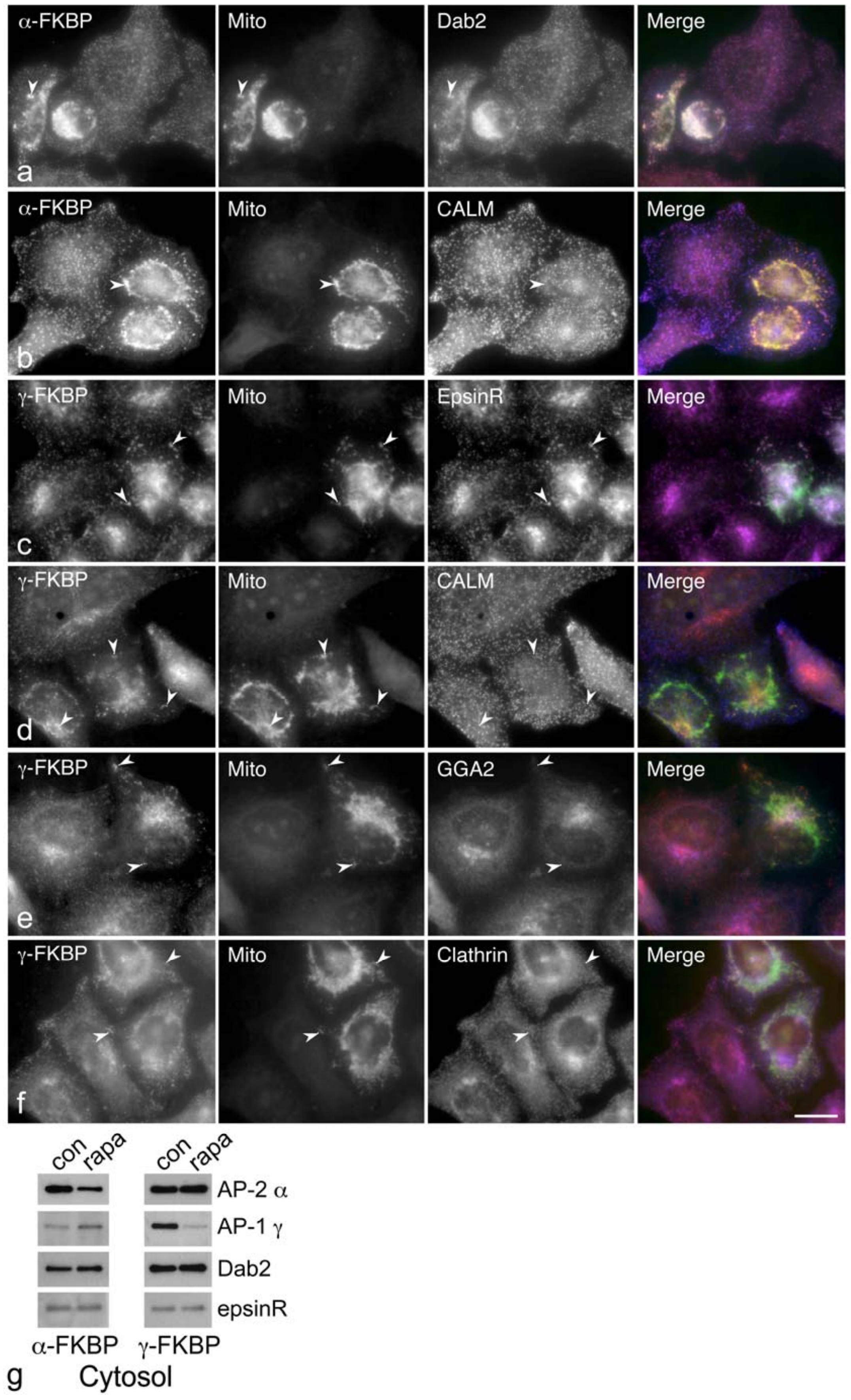

Figure S2. Effect of rerouting on other CCV components
(a-f) Immunofluorescence triple labeling. Mixed populations of cells, expressing either FKBP-tagged APs only, or coexpressing FKBP-tagged APs (red in merge) and Mito-YFP-FRB (green in merge), were depleted of the endogenous AP subunit, treated with 200 nM rapamycin for 10 min, and triple labeled for other CCV-associated proteins (blue in merge). Dab2 and epsinR follow their respective adaptors onto mitochondria, but CALM,

GGA2, and clathrin do not. Scale bar: 20 μm. g, Rerouting APs to mitochondria causes the APs themselves to be lost from cytosol, but the cytosolic pool of accessory proteins - including Dab2 and epsinR - is unaffected, indicating that even when they are relocated to mitochondria, they still cycle on and off the membrane. Although it is not clear why only some CCV-associated proteins interact with AP complexes on mitochondria, there are differences between physiological recruitment and rapamycin-induced rerouting. The AP complexes are attached to mitochondria via their flexible linkers instead of their cores (see Figure 1b), which might affect the way that some binding sites are presented; and mitochondria do not contain some of the other components that contribute to coat protein recruitment, such as phosphoinositides, small GTPases, and cargo proteins. Nevertheless, clathrin and other coat proteins readily coassemble with AP complexes in vitro, and they can also be induced to interact with APs on protein-free liposomes (Takei et al., 1998; Cell 94: 131-141). Thus, the absence of such proteins on mitochondrial membranes raises the possibility that in vivo, there may be additional regulatory mechanisms that prevent aberrant coat protein recruitment and assembly.

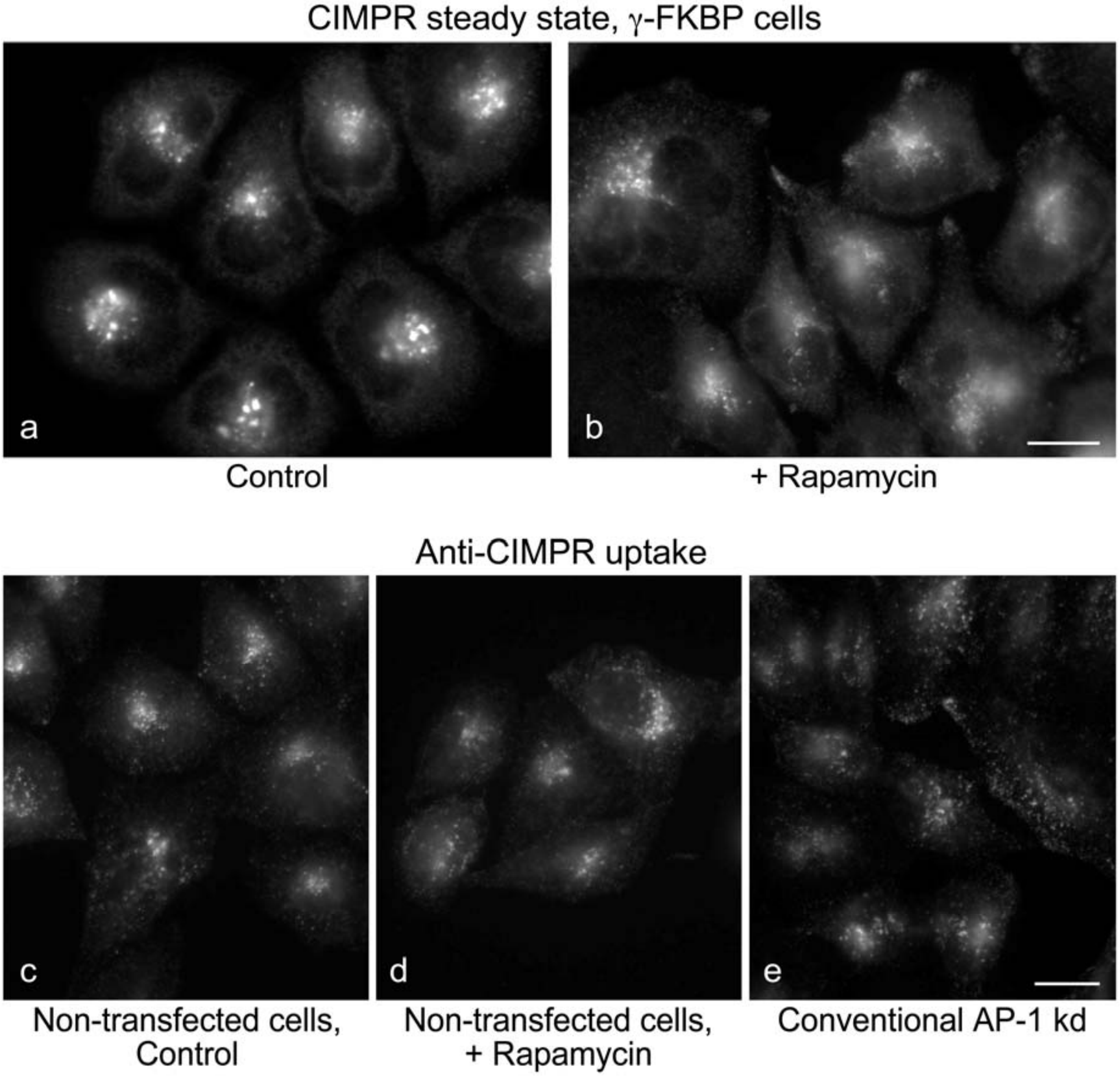

Figure S3. Localization of the CIMPR under various conditions
(a and b) Steady state distribution of the CIMPR in cells coexpressing γ-FKBP and Mito-YFP-FRB, depleted of endogenous γ and treated with rapamycin for either 0 (a) or 45 (b) min. The ratio of peripheral to juxtanuclear CIMPR is higher in the rapamycin-treated cells, although less pronounced than in an antibody uptake experiment (see Figure 4, a and b).

(c and d) Non-transfected cells were incubated with anti-CIMPR for 45 min at 37°C, either with (d) or without (c) rapamycin. The rapamycin has no effect on the localization of the endocytosed antibody. e, Cells were depleted of AP-1 γ using a conventional knockdown, then incubated with anti-CIMPR for 45 min at 37°C. The endocytosed antibody has a more peripheral distribution than in control cells, although the phenotype is less dramatic than in cells with AP-1 rerouted to mitochondria (see Figure 4, a and b). Scale bars: 20 μm.
