# Supplementary figures and images for: Rapid Inactivation of Proteins by Rapamycin-Induced Rerouting to Mitochondria
Source: Dev Cell. 2010 Feb 16;18(2-3):324–31. doi: 10.1016/j.devcel.2009.12.015 (PMC2845799; doi:10.1016/j.devcel.2009.12.015)

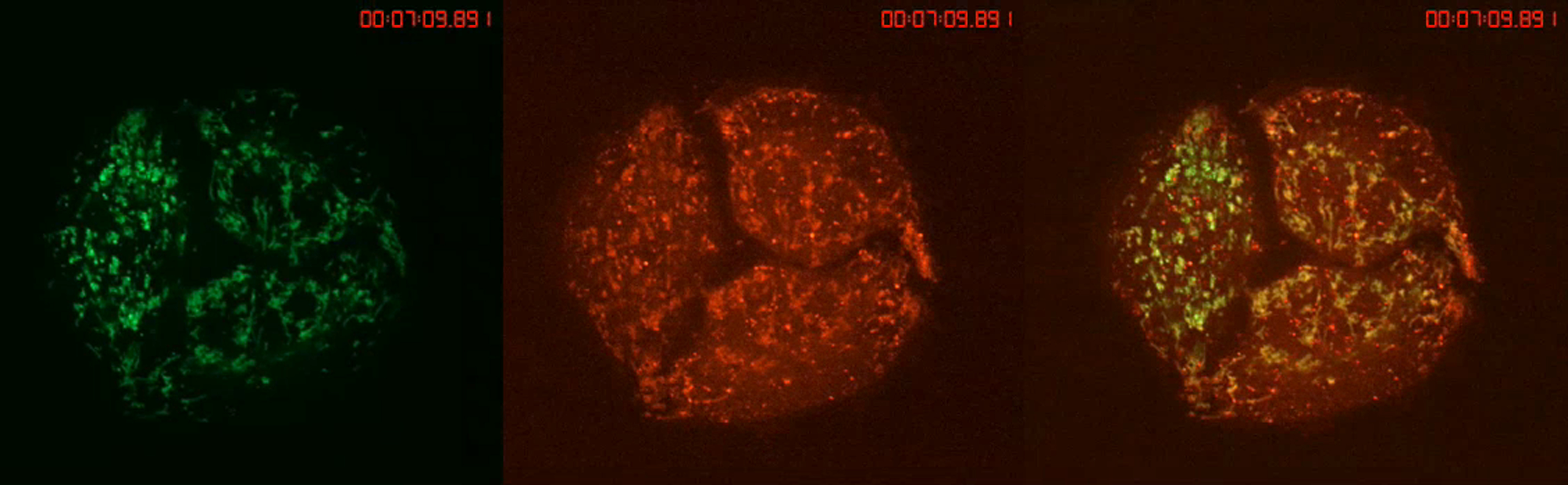

Supplement: Movie S1. Live cell imaging of rapamycin-induced rerouting of AP-2 to mitochondria — See Figure 2 for still images. [file mmc2.jpg]
